# Bone Status in a Patient with Insulin-Like Growth Factor-1 Receptor Deletion Syndrome: Bone Quality and Structure Evaluation Using Dual-Energy X-Ray Absorptiometry, Peripheral Quantitative Computed Tomography, and Quantitative Ultrasonography

**DOI:** 10.3389/fendo.2017.00227

**Published:** 2017-09-05

**Authors:** Paola Pelosi, Elisabetta Lapi, Loredana Cavalli, Alberto Verrotti, Marilena Pantaleo, Maurizio de Martino, Stefano Stagi

**Affiliations:** ^1^Department of Health Sciences, University of Florence, Anna Meyer Children’s University Hospital, Florence, Italy; ^2^Genetics and Molecular Medicine Unit, Anna Meyer Children’s University Hospital, Florence, Italy; ^3^Department of Neuroscience, Neurorehabilitation Section, University of Pisa, Pisa, Italy; ^4^Department of Paediatrics, University of L’Aquila, L’Aquila, Italy

**Keywords:** insulin-like growth factor-I receptor, insulin-like growth factor-I, bone metabolism, quantitative ultrasonography, peripheral quantitative computed tomography

## Abstract

Haploinsufficiency of the insulin-like growth factor (*IGF*)-1 receptor (*IGF1R*) gene is a rare, probably under-diagnosed, cause of short stature. However, the effects of *IGF1R* haploinsufficiency on glucose metabolism, bone status, and metabolism have rarely been investigated. We report the case of a patient referred to our center at the age of 18 months for short stature, failure to thrive, and Silver–Russell-like phenotype. Genetic analysis did not show hypomethylation of the 11p15.5 region or uniparental disomy of chromosome 7. Growth hormone (GH) stimulation tests revealed GH deficiency, whereas IGF-1 was 248 ng/mL. r-hGH treatment showed only a slight improvement (from −4.4 to −3.5 SDS). At 10 years of age, the child was re-evaluated: CGH-array identified a heterozygous *de novo* 4.92 Mb deletion in 15q26.2, including the *IGF1R* gene. Dual-energy X-ray absorptiometry showed a normal bone mineral density *z*-score, while peripheral quantitative computed tomography revealed reduced cortical and increased trabecular elements. A phalangeal bone quantitative ultrasonography showed significantly reduced amplitude-dependent speed of sound and bone transmission time values. The changes in bone architecture, quality, and metabolism in heterozygous IGF1R deletion patients, support the hypothesis that IGF-1 can be a key factor in bone modeling and accrual.

## Introduction

Insulin-like growth factor (IGF)-1 (IGF-1) and 2 (IGF-2) are major regulators of cell growth, proliferation, and death (1). IGF plays an important role in several tissues through the IGF receptor type 1 (IGF1R), a heterotetramer (α_2_β_2_) with intrinsic tyrosine kinase activity (1).

Insulin-like growth factor-1 is a crucial factor in bone formation and remodeling *via* its actions on both osteoblasts and osteoclasts, and it is involved in the development of peak bone density during growth and bone loss in senile osteoporosis (2, 3). Furthermore, IGF-1 regulates glucose metabolism by modulating insulin sensitivity and secretion (4); it is also expressed in the central nervous system and is essential for brain development (5, 6).

Haploinsufficiency of the gene encoding the *IGF1R* may be a rare and under-diagnosed cause of short stature (7, 8). Both heterozygous deletion and point-inactivating mutations of the gene can lower IGF1R mRNA and protein expression, with partial IGF-1 resistance (7, 8).

Different clinical phenotypes, including microcephaly, mental retardation, and facial dysmorphic features, were observed in patients with *IGF1R* deletion, but these phenotypes are uncommon in *IGF1R* mutations (4, 6–21). Some of the phenotypical features described in the patients are likely to be attributable to the absence of one copy of the *IGF1R* gene, but other findings are linked to the haploinsufficiency of other genes included in the deletion (7, 14, 22). Heterozygous missense and nonsense mutations of *IGF1R* show similar effects on growth and development. However, *IGF1R* mutations and deletions seem to differ *in vitro*, probably because of a dominant-negative effect of the mutation, which could decrease the number of fully functional receptors by up to 25%, whereas haploinsufficiency would theoretically lead to a 50% reduction (15).

The first *IGF1R* mutation was described by Abuzzahab et al. These authors reported the case of two patients with a history of intrauterine growth restriction (IUGR), poor postnatal growth, and the biochemical features of IGF-1 resistance (9). One patient was a compound heterozygote for point mutations in exon 2 of the *IGF1R* gene and the other carried a nonsense mutation (Arg59stop) of the same gene (9).

Walenkamp et al. described the case of a girl carrying a terminal 15.q26.2 → qter deletion. She displayed growth retardation, microcephaly, short stature, and elevated IGF-1 levels. She was treated with growth hormone (GH) with a good growth response and her final height was within the normal range (14).

Various aspects of IGF1R defects have been analyzed to date, but the effects of *IGF1R* haploinsufficiency on bone status and metabolism have not been reported.

So, in this report, we describe a female patient with a terminal deletion of chromosome 15, involving the *IGF1R* gene, who has been treated with r-hGH from the age of 4.5 years with a long-term follow-up. In this patient, we evaluated glucose metabolism and bone metabolism and status using dual-energy X-ray absorptiometry (DXA), peripheral quantitative computed tomography (pQCT), and quantitative ultrasonography (QUS).

As per institutional and national guidelines, no ethics approval was needed. Written informed consent was obtained from the parents before publication of this case report and any accompanying images.

## Case Report

The proband was a toddler referred to the Paediatric Auxoendocrinology Unit of Meyer Children’s University Hospital of Florence for short stature and failure to thrive.

The patient was the first child of healthy, unrelated parents and was born after a 36-week gestation, complicated by unexplained (IUGR), by cesarean section. No behavioral or dietary problems affected the proposita’s mother during pregnancy. The Apgar score was 7^I^-9^V^. Birth weight was 1,930 g (−1.46 SD), length 41.6 cm (−2.07 SD), and head circumference 29.2 cm (−2.16 SD).

She was breastfed, but did not catch up after birth, showing failure to thrive and suffering gastroesophageal reflux symptoms from the first months of life. Her psychomotor development was slightly delayed: she sat upright at 7 months, began to walk independently at 16 months, and began to use language at 17 months.

At 18 months of age, she was referred to our Hospital; at physical examination, she showed dysmorphic features suggestive of Silver–Russell Syndrome: a triangular face, with a prominent forehead and micrognathia, clinodactyly V, inferior limb asymmetry (near 1.5 cm), and multiple hyperpigmented lesions on the body. Her length was 67 cm (−4.27 SD), weight 6.600 kg (−3.77 SD), and head circumference 44.5 cm (−2.00 SD). Her mother’s height was 169 cm (1.09 SD) and the height of her father was 176 cm (−0.16 SD); consequently, her target height was 166.5 cm (0.67 SD). Screening blood tests, including celiac, disease serological markers, and thyroid function, were normal; basal IGF-1 was in the upper normal range (248 µg/L; 97th percentile 250 ng/mL). Bone age was delayed: 10 months at 18 months of age. She showed a normal female karyotype (46, XX), whereas the methylation study of the 11p15.5 region and the evaluation of uniparental disomy of chromosome 7 were both negative.

At the age of 4 years and 4 months, she was newly evaluated for a persistent and significant growth delay [length was 81.5 cm (−5.11 SD), weight 8.860 kg (−6.33 SD), and body mass index (BMI) 13.31 (−1.83 SD)].

An endocrine work-up was performed: free-thyroxin [(FT_4_) 1.32 ng/dL, n.v. 0.86–2.12 ng/dL], thyroid-stimulating hormone [(TSH) 3.03 μUI/dL, n.v. 0.4–4.0 μUI/dL], cortisol (11.34 µg/dL, n.v. 5–25 µg/dL), adrenocorticotropic hormone [(ACTH) 27 ng/L, n.v. 09–52 ng/L], glucose (78 mg/dL, n.v. 55–110 mg/dL), and prolactin [(PRL) 127 mUI/ml] were in the normal range. The electrolyte, venous blood gas, hemoglobin, total protein, serum albumin, coagulation profile, calcium, and phosphorous were also normal. The anti-tissue transglutaminase (tTG) test was negative.

**Figure 1:**
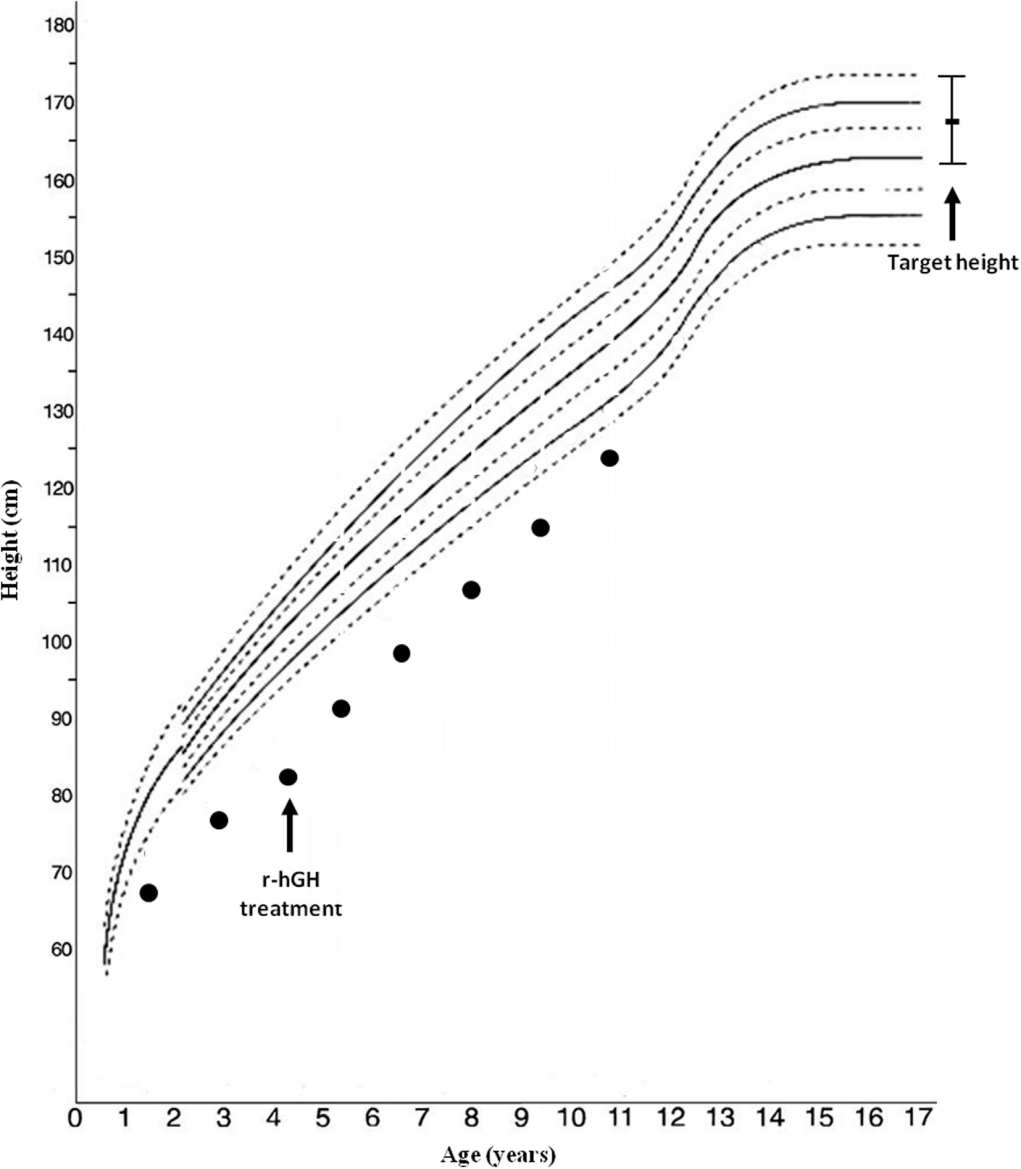
Growth chart of the patient.

Arginine [basal (GH) 1.99, peak 8.92 ng/mL] and clonidine (basal GH 0.48, peak 6.92 ng/mL) stimulation tests disclosed a GH deficiency; IGF-1 level was 243 µg/L. Bone age was significantly delayed: 1 year, 10 months at 4 years, 4 months of chronological age. r-hGH treatment was started at a dosage of 0.23 mg/kg/week; the auxological follow-up showed a slight improvement in the first year of r-hGH treatment (from −5.11 to −3.5 SD) (Figure 1).

During r-hGH therapy: IGF-I was persistently between the 90th and 97th percentile for age and sex, with glycemia, FT_4_, TSH, basal glycemia, basal insulin, and glycosylated hemoglobin (HbA1c) in normal ranges.

Therefore, in light of the unsatisfactory response to r-hGH treatment, a re-evaluation was performed at the age of 10 years and 10 months: height was 124.7 cm (−2.82 SDS), weight 21.900 kg (−2.93 SDS), BMI 14.08 (−2.14 SDS), and pubertal evaluation was B1 PH1 AH1. A re-testing of GH secretion confirmed low values of GH after arginine (basal GH 1.31, peak 7.76 ng/mL) and clonidine (basal GH 1.11, peak 7.23 ng/mL) testing; the IGF-I level was 371 µg/L. Bone age was still significantly delayed: 7 years, 11 months at 10 years, 10 months of chronological age. A new extensive endocrine work-up gave normal results. The tTG test was negative. Since the presence of HbA1c was in the upper limit of normality, we performed an oral glucose tolerance test that disclosed a reduced glucose tolerance: fasting glucose was 83 mg/dL, 2-h glucose 181 mg/dL, fasting insulin 2.19 μU/mL, peak 54.2 μU/mL 2-h insulin 54.0 μU/mL. The patient showed low leptin level (0.5 ng/mL, n.v. 1.0–12.0 ng/mL). At 11 years old, her intelligence quotient (IQ) was 108, even though the performance IQ was 85 and she exhibited some behavioral abnormalities.

### Genetic Analysis

**Figure 2:**
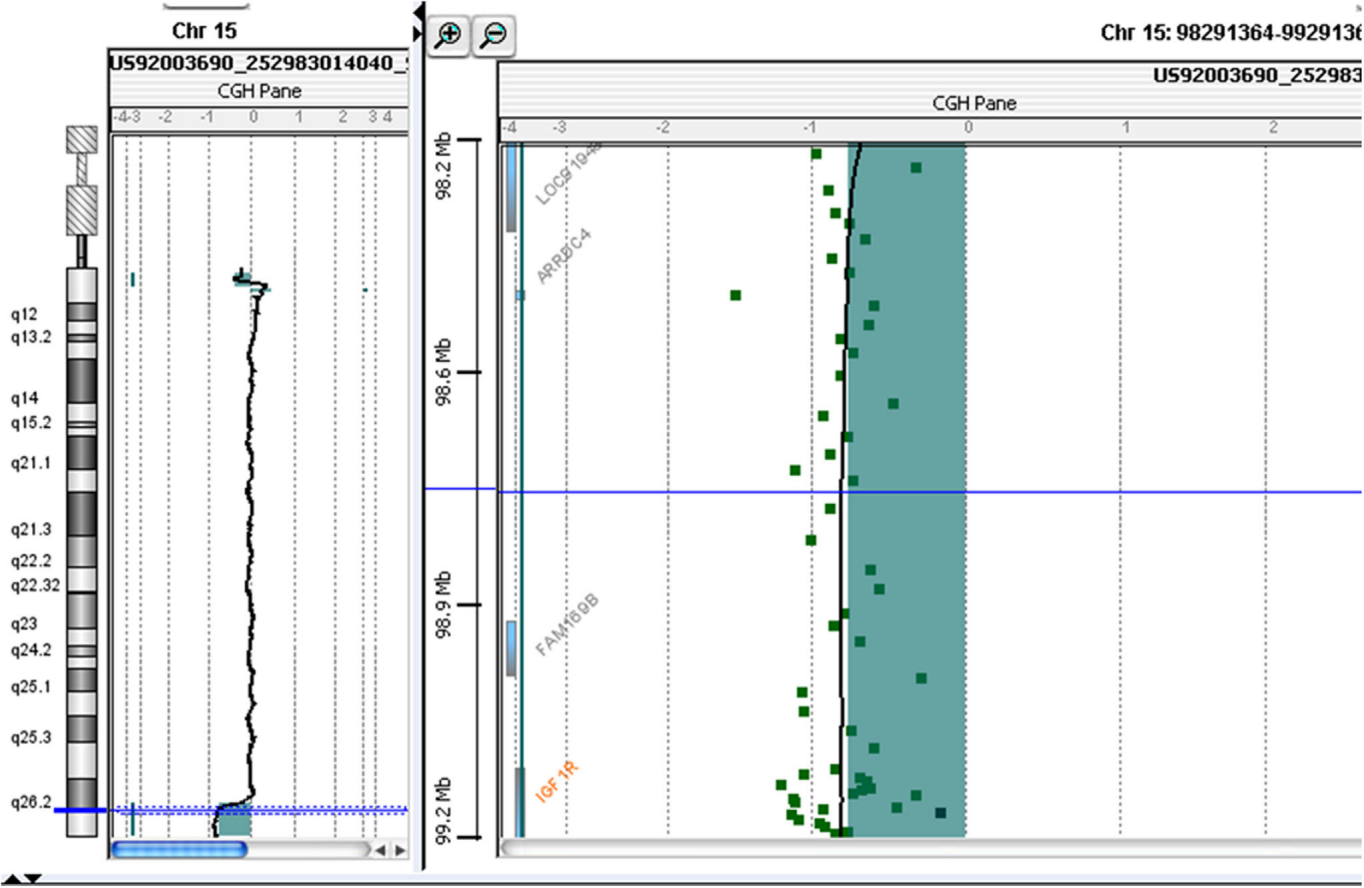
Molecular karyotyping was performed by array-CGH on the proband’s DNA using an Agilent 60 K array platform with a resolution of approximately 100 kb. Based on the physical mapping positions of the February 2009 Assembly (GRCh37/hg19) of the UCSC Genome Browser, this analysis showed a deletion of approximately 4,942 Mb that involved the 15q26.2-q26.3 region, with the breakpoint falling between 97,457,185 bp (first deleted oligomer) and 102,399,819 bp (last deleted oligomer).

At 10 years and 10 months of age, CGH-array analysis was performed using the Agilent Human Genome CGH Microarray Kit 60 K (Agilent Technologies, Santa Clara, CA, USA). The CGH-array revealed a heterozygous deletion of chromosome 15, comprising 4.942 Mbp of the terminal part of its long arm (15q26.2-q26.3) involving several genes, such as *IGF1R, ADAMTS17* (*A Disintegrin-Like and Metalloproteinase with Thrombospondin Type 1 Motif, 17*), *CERS3* (*Ceramide Synthase 3*), *ALDH1A3* (*Aldehyde Dehydrogenase 1 Family, Member A3*), and *Chondroitin Sulfate Synthase 1* (CHSY1) (Figure 2; Table 1) The deletion was not present in the parents.

**Table 1:** Genes involved in the 15q26.2-q26.3 deletion of the patient.

Gene	Possible effects on bone structure and quality
*IGF1R*	Decreased bone quality, impaired cortical density, and increased trabecular density
*ADAMTS17*	BDNA. Homozygous mutation in the *ADAMTS17* gene caused Weill–Marchesani-like syndrome l
*CERS3*	BDNA. Homozygous mutation in the *CERS3* gene caused a form of congenital ichthyosis
*ALDH1A3*	BDNA. Homozygous mutation in the *ALDH1A3* gene caused a form of microphthalmia
*CHSY1*	BDNA. Mice lacking Chsy1 display chondrodysplasia and decreased bone density

*IGF1R, insulin-like growth factor I receptor; ADAMTS17, a disintegrin-like and metalloproteinase with thrombospondin type 1 motif, 17; CERS3, ceramide synthase 3; ALDH1A3, aldehyde dehydrogenase 1 family, member A3; CHSY1, chondroitin sulfate synthase 1; BDNA, bone data not available*.

### Bone Density and Structure Evaluation

At the age of 10 years and 11 months, the patient underwent an evaluation of bone metabolism, density, and structure. Bone mineral density (BMD, g/cm^2^) was measured by DXA at the lumbar spine (L2–L4) (Delphi-A System, Hologic, Inc., Waltham, MA, USA) and expressed as *z*-scores. To estimate the volumetric density (bone mineral apparent density or BMAD), we used the formula of Kröger et al. (23). The BMD *z*-score, corrected for height, was 0.67: BMD at the lumbar spine was 0.631 g/cm^2^ and the bone mineral content was 22.27 g.

**Figure 3:**
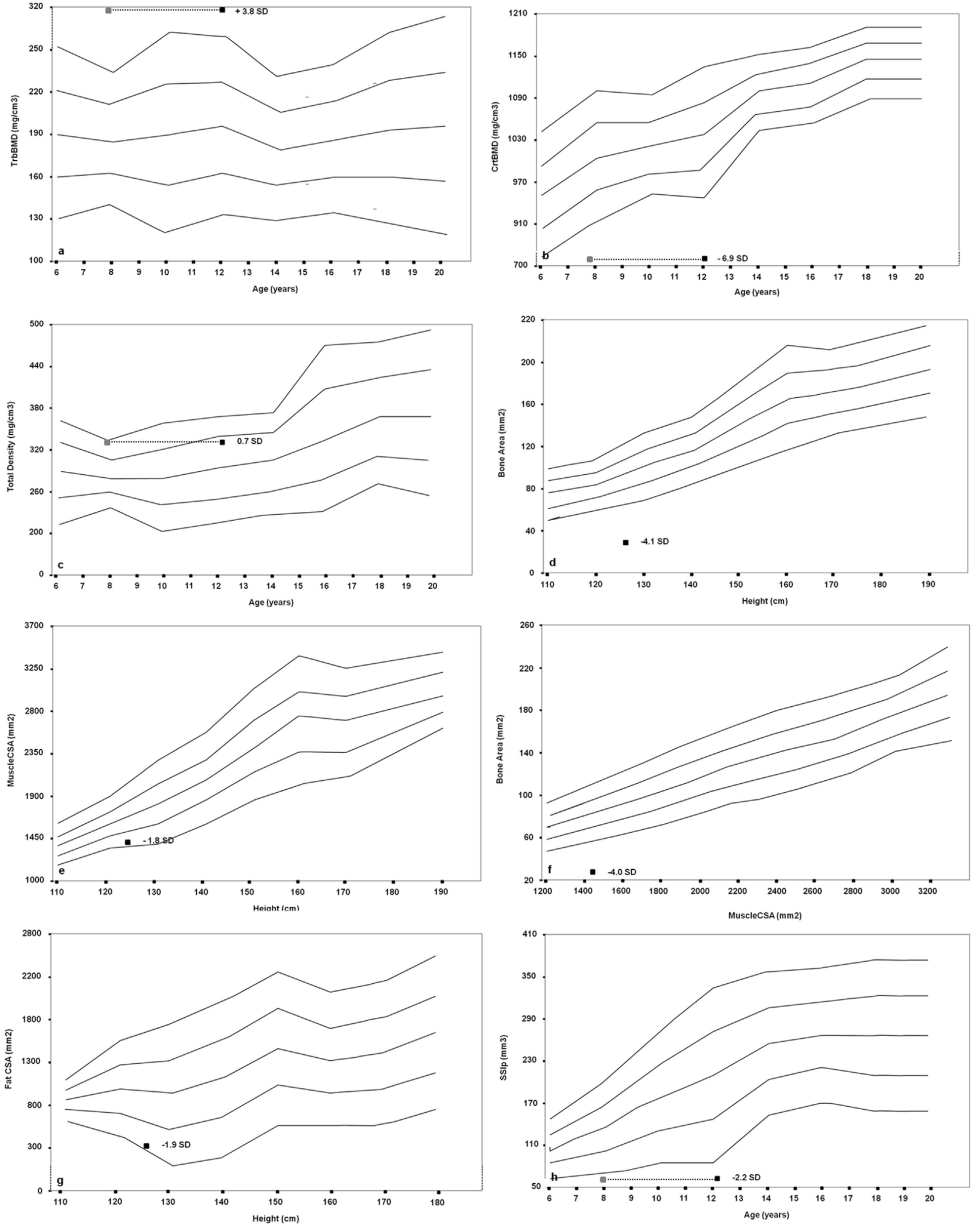
Cross-sectional evaluation of trabecular bone mineral density (TrabBMD) **(A)**, cortical bone mineral density (CrtBMD) **(B)**, total density corrected for age **(C)**, bone area corrected for height **(D)**, muscle cross-sectional area (MuscleCSA) corrected for height **(E)**, bone area corrected for MuscleCSA **(F)**, fat cross-sectional area (FatCSA) corrected for height **(G)**, and density-weighted polar section modulus (SSIp) **(H)**. The gray squares in the panels **(A,B,C,H)** represent the bone age-adjusted values.

Furthermore, we performed a pQCT of the left (non-dominant) radius at sites 4 and 66% using a Norland-Stratec XCT 3000 scanner (Stratec Medical, Pforzheim, Germany). As for the growth retardation of the patient, all bone size-dependent parameters (Total, Cortical, and MuscleCSA) were corrected for height (24, 25). We disclosed an imbalance between the trabecular and cortical bone, with an augmented trabecular component (318.4 mg/cm^3^, *z*-score 3.8) and a very low cortical density (727.8 mg/cm^3^, *z*-score −6.9) in relation to the age. The proband showed a normal total density value (321.3 mg/cm^3^, *z*-score 0.7) and a significantly reduced bone area for muscle area (31.2 mm^2^, *z*-score −4.0) and for height (28.9 mm^2^, *z*-score −4.1). The SSI polar (62.5 mm^3^, *z*-score −2.2) was significantly reduced. Fat and muscle components were also poorly represented (304 mm^2^, *z*-score −1.8; and 1,376 mm^2^, *z*-score −1.9, respectively) (Figures 3A–H).

Finally, the bone quality status was evaluated with a DBM Sonic 1200 device (IGEA Bone Profiler, Carpi, Italy) (24). The evaluation showed a very low amplitude-dependent speed of sound (AD-SoS, 1,791 m/s; *z*-score −3.85) and bone transmission time (BTT, 0.78 µs; *z*-score −2.18) values. Since bone size could also influence QUS parameters (26), we created a height-adjusted *z*-score for AD-SoS.

The study of the bone metabolism showed a low 25(OH) vitamin D [25(OH)D] level (14.3 ng/mL, n.v. >20 ng/mL) and a moderately high parathyroid hormone level (51 pg/mL; n.v. <43 pg/mL). Total protein, serum albumin, calcium, and phosphorous levels were normal; however, osteocalcin (34.3 mg/ml; n.v. 55–135 mg/ml), bone alkaline phosphatase (30 IU/L; n.v. 39.4–346.1 IU/L), and urinary deoxypyridinoline concentrations (23.45 nM/mM creatinine; n.v. 30.3–54.7 nM/mM creatinine) were lower than reference values for sex and age.

## Discussion

We reported the case of a heterozygous, *de novo* 15q26.2-26.3 deletion involving the *IGF1R* gene. As in other previous reported cases of *IGF1R* deletion, our patient had a history of unexplained IUGR and severe short stature without catch-up growth (8, 10–14).

A recent study found that *IGF1R* haploinsufficiency was present in 2 out of 100 short small for gestational age (SGA) children with persistent short stature who benefited from GH therapy with moderate catch-up growth. The authors suggest that IUGR, microcephaly, micrognathia, relatively high IGF-1 levels, and developmental delays are the main predictors of IGF1R deletion (15). Our patient, treated with GH linear growth showed only a slight improvement, although she still remained persistently below her target height (Figure 1). Treatment response seems to be variable across patients, but all cases reported in the literature benefited from therapy, through the increase of GH and IGF-1 levels and most likely overcoming IGF-1 partial resistance (14, 15, 27).

Furthermore, our study provides very interesting data about bone structure and metabolism in *IGF1R* deletion patients, assessed by using three different approaches and sites scanned: DXA in lumbar spine, typically rich in trabecular bone (28); pQCT at radius, distinguishing cortical and trabecular components, as well as fat and muscle areas (24); quantitative ultrasonography at non-dominant hand phalanges, providing information not only on the density but also on structure and mechanical properties of the segment in question, whose composition is similar to femoral one (24, 29).

The data obtained in this *IGF1R* haploinsufficient patient seem to suggest an impaired cortical bone density (low *z*-scores at pQCT) and an increased trabecular component, despite normal BMD evaluated by DXA. As we described in a previous work (29), in fact, pQCT allows us to differentiate trabecular and cortical components, unlike the projective methods, such as DXA. The impaired bone quality of cortical component, well represented in phalanges, also resulted in the low *z*-score values related to density and stiffness measured at QUS. Moreover, pQCT has the advantage of measuring the real density of bone in a given volume without the superposition of other tissues, reducing the effect of auxological parameters, such as height and BMI. Therefore, the apparently normal outcome of lumbar DXA *z*-score is probably due to the prevalence of trabecular bone in vertebral site, component that may be less affected in IGF-1 disorders. The impairment of cortical compartment is instead shown by radial pQCT, which assess it separately from trabecular one, and by QUS, which evaluate a site where cortical bone is well represented, i.e., phalanges.

Several studies have demonstrated that humans lacking a functional *IGF-1* gene suffer from severe osteopenia (30); however, mice that are deficient in liver-derived IGF-1 (LID), acid labile subunit (ALS) knockout mice (KO), and double gene disruption LID + ALSKO mice have a reduced femoral periosteal circumference, a smaller cross-sectional area, and a thinner cortical bone, when compared to control mice (31), which may be partially explained by the dramatic growth retardation in IGF-1-deficient mice (32). However, altered parameters were observed in both the trabecular and cortical compartments of IGF-I-null mice femurs, compared to wild-type mice (32). In rats, combined rhGH and rhIGF-1 treatment appeared to stimulate cortical bone mass, as evaluated by pQCT, more than rhGH alone does (33). Nevertheless, another study conducted in young adult mice with an IGF-I gene deletion showed significant alterations in bone mass and bone structure in both the cortical and trabecular compartments (32). Previous studies in IGF-I-deficient mice of a different background showed a decrease in cortical bone formation but an increase of several trabecular parameters in the tibia (34, 35). These data indicate that circulating IGF-1 is critical for bone modeling, quality, and structure, and it has been hypothesized that bone regional differences in response to IGF-I deficiency might be a consequence of the dual effect of IGF-1 on both osteoblastogenesis and osteoclastogenesis (36).

Circulating IGF-1 exerts its anabolic effects on the periosteal surface. Studies have shown that circulating IGF-1 stimulates periosteal bone growth along the cortex (37), whereas reduced serum levels of IGF-1 in mice lacking liver-specific IGF-1 were associated with impaired periosteal apposition, leading to the development of slender bones during growth (38). When subjected to a loading regimen, periosteal bone formation was substantially elevated in the IGF-1-overexpressing mice but not in wild-type littermates (39), suggesting that circulating IGF-1 enhances bone responses to mechanical loading.

We can observe that the haploinsufficiency of *IGF1R* may cause an imbalance of bone quality, structure, and modeling, probably leading to inferior mechanical properties and an increased risk of fracture, as showed by the SSIp value.

However, although IGF-1 undoubtedly affects GH action, it is now clear that: GH can have direct effects on multiple tissues; IGF-1 has endocrine, paracrine, and autocrine effects that are, in part, GH-independent; and GH and IGF-1 are likely to have overlapping, counteractive, and/or collaborative effects (40). However, we cannot know the effects of the performed r-hGH treatment on the bone quality and structure of our patient. More data are necessary to better understand this aspect in patients with *IGF1R* deletion.

Using current knowledge, it is likely that the only other gene involved in this patient’s deletion that could have affected the patient’s bone is *CHSY1*. Mice lacking Chsy1 for homozygous mutations display chondrodysplasia and decreased bone density, but no such data are available for humans.

Furthermore, IGF-1 also exerts an anabolic action on muscle, increasing the protein synthesis and decreasing the protein breakdown (41, 42). In fact, IGF-1 elicits skeletal muscle cell proliferation and myocytes differentiation (42). These data may explain the poorly represented muscle component in our patient.

Finally, another interesting aspect is the role of the IGF-system on glucose metabolism and the possible effects of *IGF1R* haploinsuficiency on carbohydrate homeostasis, as shown by our case report. The *IGF1R* gene has sequence homology with the insulin receptor gene; both are transmembrane tyrosine kinase receptors. IGF-I also has structural homology with pro-insulin and has insulin-like metabolic effects, while GH has some effects that are antagonistic to those of insulin (43). IGF-1 is important in maintaining beta-cell mass by stimulating their proliferation and can enhance peripheral insulin sensitivity (44). Mohn et al. studied four members of a family carrying a novel nonsense mutation of the IGF1R gene. The defect was associated with a variable impairment of prenatal and postnatal growth. The authors also reported alterations in carbohydrate metabolism, ranging from normal glucose tolerance in the presence of insulin resistance to IGT and fasting hyperglycemia in association with both insulin resistance and impaired beta-cell function (4).

In conclusions, our study showed the presence of changes in bone architecture, quality, and metabolism in a heterozygous *IGF1R* deletion patient. The changes in bone metabolism due to the lack of action of IGF-1, a key in bone modeling and accrual, as occurring in a heterozygous IGF1R deletion patient, can be well evaluated through three different techniques (DXA, p-QCT, and QUS) assessing its effects on bone density and quality.

## Author Contributions

PP carried out the endocrinological evaluation, conceived the study, and participated in its design. EL carried out the clinical genetic diagnosis, conceived the study, and participated in its design. LC and AV participated in the endocrinological evaluation and in the design of the study. MM participated in the endocrinological evaluation and in the coordination of the study. SS carried out the endocrinological evaluation, conceived the study, and participated in its design and coordination. All authors have read and approved the final manuscript.

## Conflict of Interest Statement

The authors do not have any financial or non-financial competing interests in relation to this manuscript.
